# The influence of palm oil additives on the pour point and wax deposition tendencies of Chenor crude oil

**DOI:** 10.1007/s13202-021-01316-w

**Published:** 2021-10-12

**Authors:** Thevaruban Ragunathan, Colin D. Wood, Hazlina Husin

**Affiliations:** 1 Universiti Teknologi PETRONAS, Bandar Seri Iskandar, 31750 Seri Iskandar, Perak Darul Ridzuan Malaysia; 2 CSIRO Energy Business Unit, 26 Dick Perry Avenue, Kensington, WA 6102 Australia

**Keywords:** Paraffin wax, Chemical wax inhibitors, Pour point depressant, Palm oil inhibitors, Cold finger test, Pour point test

## Abstract

One of the major concerns during the production of crude oil especially in tropical waters is the deposition of wax on to the walls of the pipeline. This is due to the low seabed temperatures which can be below the wax appearance temperature (WAT) which leads to wax depositing out through molecular diffusion. Currently, there are many methods to prevent and remedy wax deposition but most of these solutions pose a serious environmental threat and are expensive to produce. Hence, this research investigated the use of an organic and cheaper alternative by utilizing synthetic fatty acid esters such as oleic acid which has shown promising results in reducing the pour point of waxy crude oils. The solution that was used was of palm oil origin, crude palm oil (CPO) and crude palm kernel oil (CPKO) and was subsequently compared with the pour point depressant and wax inhibition efficiency of the current industry used inhibitors utilizing the SETA Pour Point and Cloud Point as well as the cold finger apparatus. It was observed that the palm oil inhibitors were highly effective at 1 wt.% due to the high composition of oleic acid present portraying a similar result to Triethanolamine (TEA) while Ethylene Vinyl Acetate (EVA) performed best at low concentration of 0.1 wt.% but deteriorates significantly as the concentration increases due to the polar end agglomerating among itself.

## Introduction

With the current COVID 19 pandemic and significantly low demand of crude oil, the production of crude oil has decreased in order to reduce the loss due to the low oil price. However, demand remains for the production of crude oil due to the high energy density and versality as compared to other source of energy (Agency 2018). Therefore, for oil producing countries and organizations, the extraction, transportation as well as storage of crude oil is significant in guaranteeing a safe rate of return in investment.

There are a number of flow assurances issues that must be considered to prevent blockages and other difficulties during the production of crude oil. Among the flow assurance issues include hydrate formation, paraffin wax deposition, asphaltenes, erosion and corrosion (Arnold and Stewart 1999). However, in warm waters like Malaysia, the main flow assurance issue is the deposition of paraffin wax as compared to the other listed flow assurance issues (Anisuzzaman et al. 2018; Lim et al. 2018).

Paraffin waxes deposits on to the pipeline wall particularly when the temperature of the crude oil is below the Wax Appearance Temperature (WAT) or when there is a temperature differential between the crude oil and the pipeline(dos Santos et al. 2004; Hosseinipour et al. 2016; Jang et al. 2007; Ridzuan et al., 2014; Wei et al. 2015; Zheng et al. 2016). WAT is defined as the temperature where the first wax crystals start to form during the cooling of the crude oil (Aiyejina et al. 2011). Wax deposition then occurs when the long *n*-paraffin chains in the crude oil begins to crystallize and agglomerate causing it to adhere to the cold pipeline wall. This causes the decrease in the inner pipeline diameter, therefore increasing the pressure drop along the pipeline, hindering the production of oil. Hence, oil producing companies prefer to ensure that the crude oil temperature in the pipeline is above the WAT by utilizing current insulation, mechanical, thermal or chemical technologies (Aiyejina et al. 2011; Hilbert 2011).

Among the inhibition technologies, the utilization of chemical inhibitors is preferred due to the lower operating cost and maintenance. Chemical inhibitors operate by decreasing the pour point of the crude oil, augmenting the crude oil flowability and reducing the agglomeration of the wax crystals (Ferworn et al. 1997; Tung et al. 2001). Though, chemical inhibitors presently used in the industry has a substantial impact on the operating cost of the pipeline as well as possess an environmental threat if there is a spill as it is not biodegradable. From the research conducted by Akinyemi et al. (2018) where the authors utilized plant seed oils such as jatropha (JSO), rubber (RSO) and castor (CSO) as potential chemical additives in Nigerian waxy crude oil. The results revealed that the plant seed oils were able to lower the deposition of wax as effective as commercial inhibitors (ethylene–vinyl acetate). The authors then further established in their research that the elevated composition of oleic acid in the plant seed oils are the main element to the high inhibition efficiency (Akinyemi et al. 2016, 2018). Moreover, authors such as Patel et al. (2017), Soni et al. (2010) and Hafiz and Khidr (2007) have further demonstrated that oleic-based polymers can potentially be excellent chemical inhibitors where the authors utilized several esters of oleic acid as polymeric flow improvers by evaluating the rheological modifying properties. The authors then established that at elevated concentrations, the synthesized flow improvers are exceptional pour point depressants which reduces the apparent viscosity, plastic viscosity as well as the yield value of the Langhnaj crude oil (Hafiz and Khidr 2007; Patel et al. 2017; Soni et al. 2010).

In addition, chemical inhibitors utilized presently are usually polymer based which consist of two groups, polar and non-polar. The polar group works by intervening with the crystallization process including altering the morphology of the crude oil. The non-polar group of the polymer operates by interlocking the wax molecule and the inhibitor molecule together (Machado et al. 2001). The polymer inhibitors are able to adsorb onto the surface of the paraffin crystals and decreases the growth rate and crystal nucleation of the paraffin waxes. For instance, polyethylene vinyl acetate (EVA) and triethanolamine (TEA) are commercially used polymers which are able to coalesce and intermingle with wax crystals and impedes the growth of the crystals (Anisuzzaman et al. 2017; Hilbert 2010; Ridzuan et al. 2014). Therefore, this study will investigate the effectiveness of palm oil additives in reducing the pour point of crude oil in comparison to commercially used chemical inhibitors such as polyethylene vinyl acetate (EVA) and triethanolamine (TEA) using the SETA Cloud Point and Pour Point apparatus. Also, a cold finger test was conducted using Chenor crude oil in the presence of Crude Palm Oil (CPO), Crude Palm Kernel Oil (CPKO), TEA and EVA under dynamic conditions to support the findings obtained from the pour point test. It should be noted that this paper is the continuation of Ragunathan et al. (2020a; b). Table 1 portrays the composition of fatty acids in CPO and CPKO.

**Table 1:** Composition of fatty acids in CPO and CPKO (Malaysian Palm Oil Council 2012)

Fatty acid	Lipid number	CPO, %	CPKO, %
Capric acid	C_10:0_	–	3.7
Lauric acid	C_12:0_	0.5	15.3
Myristic acid	C_14:0_	1.5	15.6
Palmitic acid	C_16:0_	34.5	7.8
Palmitoleic acid	C_16:1_	0.4	–
Stearic acid	C_18:0_	5.4	2
Oleic acid	C_18:1_	44.1	48.2
Linoleic acid	C_18:2_	12.5	2.7
α-Linolenic acid	C_18:3_	0.6	–
Arachidic acid	C_20:0_	0.5	–
Caproic acid	C_6:0_	–	0.3
Caprylic acid	C_8:0_	–	4.4
9,10-Dihydroxystearic	–	–	–

## Methodology

### Materials

The palm oil (CPO) and palm kernel oil (CPKO) were obtained from United Plantation. The crude oil used from the Chenor oil field while the triethanolamine (TEA) and ethylene vinyl acetate (EVA) were purchased from Sigma Aldrich.

### Characterization of the crude oil sample

The physical as well as the chemical properties of the crude oil are as shown in Table 2. The density, specific gravity as well as the API gravity are obtained using densitometer. The wax appearance temperature (WAT) and the pour point of the crude oil were obtained using the SETA pour point and cloud point apparatus. The wax content of the crude oil was obtained using the wax content analyzer.

**Table 2:** Physical and chemical properties of crude oil sample

Chemical properties	Crude oil
Density	0.8033 g/cm^3^
API gravity	44.65°API
Wax appearance temperature/ cloud point	35 °C
Pour point	10 °C
Wax content (wt.%)	8.26

### Pour point test

The pour point of the Malaysian crude oil was analyzed utilizing the ASTM D97-12 (SETA Cloud and Pour Point) (Astm 2015). The pour point of any crude oil is the minimum temperature where the movement of crude oil can be observed under constant temperature and pressure. The pour point temperature of the crude oil is necessary to be used as a reference for the cold finger as well as to investigate the efficiency of the pour point depressants. Many studies have portrayed that the molecular weight distribution and wax content are the main factor that effects the pour point of the crude oil (Tung et al. 2001). The chemical additive utilized must have the ability to alter the crystalline state of wax during the cooling process. The first chemical group is classified as polymers and copolymers, which functions excellent in a low water content crude by inhibiting or altering the wax and crystal growth. The other chemical group is surfactants which is highly efficient in the presence of water by water-wetting the flowline or pipeline (Jennings & Breitigam 2010).

**Fig. 1:**
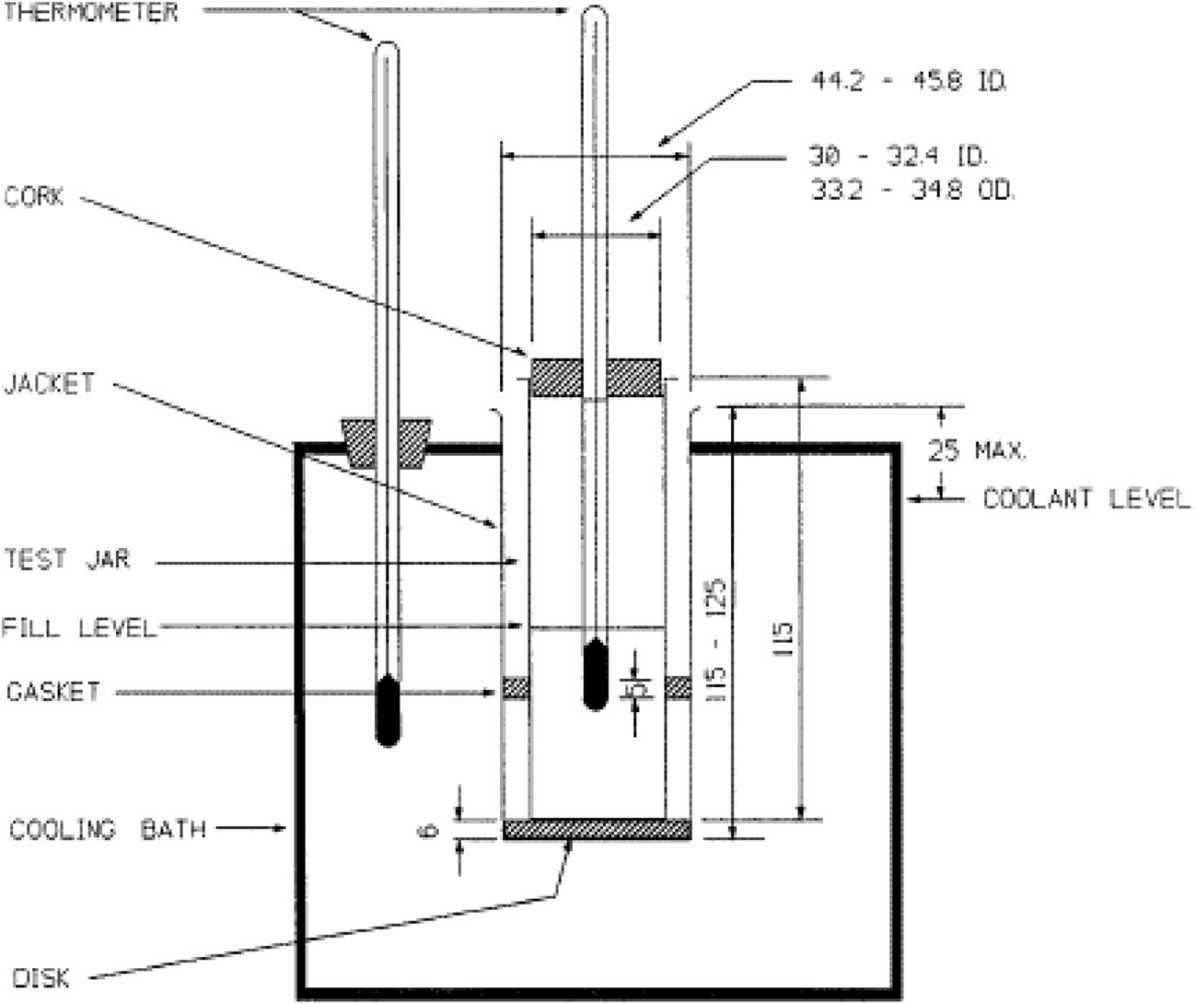
Schematics of pour point apparatus (Siddig and Younis 2010)

**Fig. 2:**
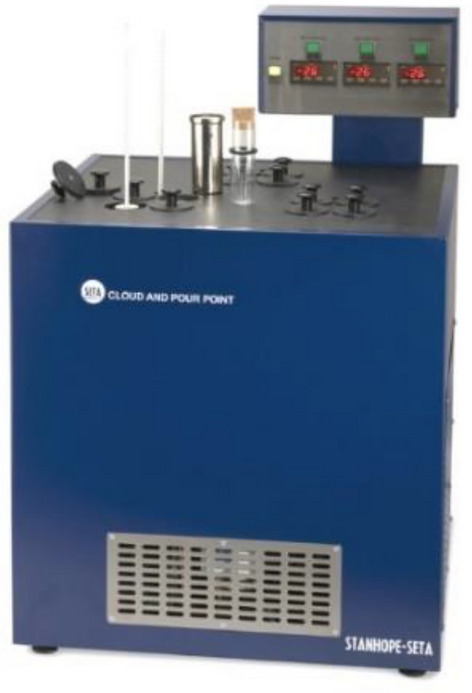
Image of SETA cloud and pour point

There are two types of pour point, the upper pour point and the lower pour point: The upper pour point is obtained after the crude oil has undergone a procedure to enhance the wax crystal gelation as well as solidification. The lower pour point is obtained when the crude oil undergoes a procedure to delay the wax crystal gelation as well as solidification. The pour points depend on the wax content of the crude oil. As observed from the research performed by Sharma and et al., the higher the pour point temperature, the higher the wax content and vice versa (Sharma et al. 2016). The pour point temperature obtained from the crude oil tested from the authors ranges from 35 °C till 51 °C (Sharma et al. 2016). The setup of the experiment is as shown in Figs. 1 and 2.

Firstly, the crude oil was heated at 70 °C for 10 h to remove the thermal history and dissolve the wax that has precipitated. Then, the crude oil was transferred into the test jar up until the marked level where the test jar was then closed using a cork carrying a thermometer. The cork and thermometer were ensured to fit tightly. The thermometer was immersed to about 5 mm below the surface of the crude oil. Next, the specimen was then heated to a temperature 10 °C above the expected cloud point without stirring (minimum 45 °C) in a water bath that was maintained at a temperature of 12 °C above the expected pour point temperature (minimum 48 °C). The test jar was then placed in another water bath that is 0 °C. Also, the test jar was placed in a jacket and to avoid the test jar from touching the walls of the jacket, the use of gasket and disk is necessary. As the crude oil was allowed to cool, great care was taken to avoid any agitation to avoid low and erroneous reading. At every decrease in oil temperature by 3 °C, the test jar was removed, and the test jar was tilted to examine the movement of the crude oil. The presence of crude oil movement will lead to the test jar being immediately placed back in the jacket, and procedure is repeated until the absence of crude oil movement is observed. Once the crude oil movement has halted, the temperature was recorded, and the experiment is repeated to obtain a consistent reading.

The experiment was then repeated in the presence of additives of TEA, EVA, CPO and CPKO at a concentration of 0.1 wt.%, 0.5 wt.%, 1 wt.%, 5 wt.% and 10 wt.%. The experiment was conducted to determine if the presence of additives aids in reducing the pour point of the crude oil.

### Cold finger test

**Fig. 3:**
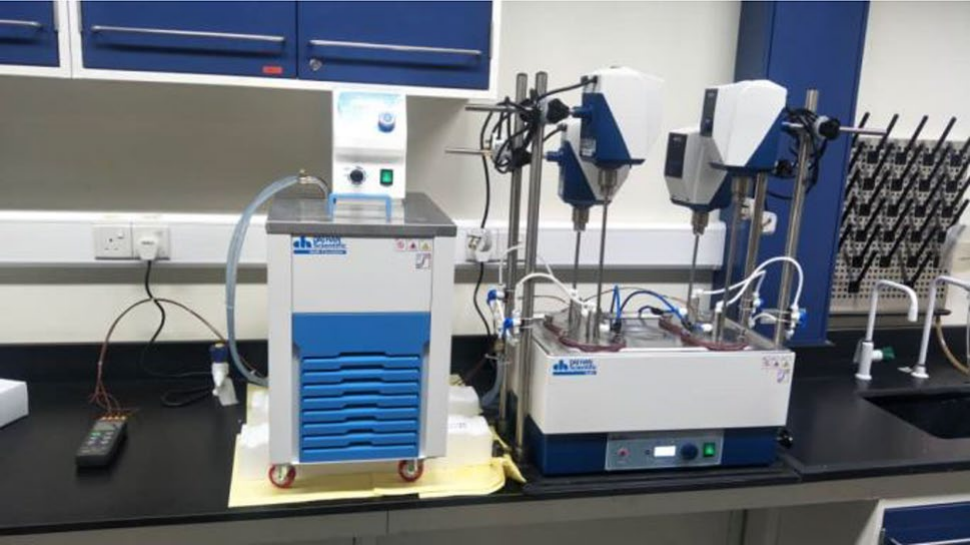
Image of cold finger apparatus

The oil tank was filled with 300 ml of the sample crude oil. The bulk oil temperature was maintained below the WAT of the crude oil at 25 °C. The cold finger was set at 5 °C to produce a temperature gradient in the sample. An overhead stirrer was used to induce sample mixing. After 2 h, the cold finger was removed, and the wax deposit was weighed. The experiment was conducted for 6 h and then extended time of 24 h to investigate the wax deposition of different ageing time. The experiment was then repeated using the crude oil in the presence of TEA, EVA, CPO and CPKO, respectively, at various concentration. Figure 3 shows the image of the cold finger apparatus used.

The Paraffin-Inhibition Efficiency (PIE) of each chemical additive was then determined using the following equation:

1 $${\text{PIE}} = \frac{{W_{{\text{p}}} - W_{{\text{a}}} }}{{W_{{\text{p}}} }} \times 100\%$$

where *W*_p_ = mass of deposited paraffin wax in the absence of inhibitors, g; *W*_a_ = mass of deposited paraffin wax in the presence of chemical additives, g.

## Results and discussion

### Pour point test

**Fig. 4:**
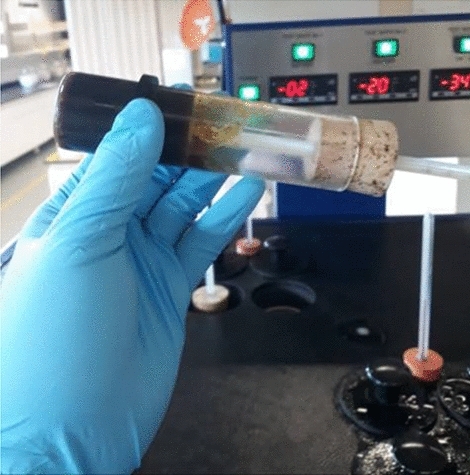
Image of crude oil at pour point temperature

The pour point of the crude oil as well as the pour point of the crude oil in the presence of various inhibitors were obtained after concluding that the crude oil did not flow after being tilted horizontally. Figure 4 shows the crude oil at pour point conditions.

The results of the test are as shown in Table 3 whereby, / sign indicates that the crude oil can still flow meanwhile X sign indicates that the crude oil stopped flowing when diverted horizontally for 3 to 5 s.

**Table 3:** Results of pour point test

Temperature, °C	Crude Oil Only	Crude Oil + EVA (%)					Crude Oil + TEA (%)					Crude Oil + CPO (%)					Crude Oil + CPKO (%)				
		0.1	0.5	1	5	10	0.1	0.5	1	5	10	0.1	0.5	1	5	10	0.1	0.5	1	5	10
24	/	/	/	/	/	/	/	/	/	/	/	/	/	/	/	/	/	/	/	/	/
22	/	/	/	/	/	/	/	/	/	/	/	/	/	/	/	/	/	/	/	/	/
20	/	/	/	/	/	X	/	/	/	/	/	/	/	/	/	/	/	/	/	/	/
18	/	/	/	/	/	X	/	/	/	/	/	/	/	/	/	/	/	/	/	/	/
16	/	/	/	/	X	X	/	/	/	/	/	/	/	/	/	/	/	/	/	/	/
14	/	/	/	/	X	X	/	/	/	/	/	/	/	/	/	/	/	/	/	/	/
12	/	/	/	/	X	X	/	/	/	/	/	/	/	/	/	/	/	/	/	/	/
10	X	/	/	/	X	X	/	/	/	/	/	/	/	/	/	/	/	/	/	/	/
8	X	/	/	/	X	X	/	/	/	/	/	/	/	/	/	/	/	/	/	/	/
6	X	/	/	/	X	X	X	/	/	/	/	/	/	/	/	/	/	/	/	/	/
4	X	/	/	X	X	X	X	/	/	/	X	/	/	/	/	/	/	/	/	/	/
2	X	/	/	X	X	X	X	X	/	/	X	/	/	/	/	/	/	/	/	/	/
0	X	/	/	X	X	X	X	X	/	/	X	/	/	/	/	X	/	/	/	/	X
− 2	X	/	/	X	X	X	X	X	/	/	X	/	/	/	/	X	X	/	/	/	X
− 4	X	/	/	X	X	X	X	X	X	X	X	X	/	/	X	X	X	/	/	X	X
− 6	X	X	X	X	X	X	X	X	X	X	X	X	X	/	X	X	X	X	/	X	X
− 8	X	X	X	X	X	X	X	X	X	X	X	X	X	X	X	X	X	X	X	X	X

/ = Flowing; X = Not flowing

From Table 3, the pour point of the crude oil is at 10 °C, in the presence of low concentration of EVA, to be specific at 0.1% and 0.5% concentration, the pour point of the crude oil reduces to − 6 °C but then increases as the concentration of EVA increases as portrayed in the presence of 1%, 5% and 10% EVA at 4 °C, 16 °C and 20 °C, respectively. Meanwhile, a slightly different trend was observed in the presence of TEA inhibitor where in the presence of 0.1% concentration, the pour point of the crude oil decreases to 6 °C, significantly higher when compared to EVA at the similar concentration. The pour point then was observed to decreased as the TEA concentration increases. At 0.5%, 1% and 5% TEA concentration, the pour point of the crude oil is recorded to be at 2 °C, − 4 °C and − 4 °C, respectively. The pour point of the crude oil in the presence of TEA then increases to 4 °C at 10% concentration of TEA.

In addition, when the pour point of the crude oil was tested in the presence of 0.1% CPO, the temperature when the crude oil stopped flowing was observed to be at − 4 °C. However, as the concentration of CPO increases, the pour point of the crude oil decreases as observed at 0.5% and 1% concentration of CPO where the pour point was observed to be at − 6 °C and − 8 °C, respectively. When the CPO concentration is further increased, the pour point of the crude oil can be seen to increase. Take instance, in the presence of 5% and 10% CPO concentration, the pour point was observed to be at − 4 °C and 0 °C, respectively. In terms of CPKO, at 0.1% concentration, the pour point of the crude oil was observed to be at − 2 °C and as the concentration of CPKO increased, the pour point decreased up to 1% concentration. At 0.5% and 1% concentration, the pour point of the crude oil was observed to be at − 6 °C and − 8 °C, respectively. As the concentration increased above 1% concentration, the pour point can be seen to increase to where at 5% concentration, the pour point was observed to be at − 4 °C while at 10% concentration, the pour point was observed to be at 0 °C.

Thus, it can be concluded at low concentrations, all the inhibitors tested are able to reduce the pour point of the crude oil but a critical concentration exists where when the concentration used surpasses the critical concentration, the inhibitors losses their ability to reduce the pour point of the crude oil and may act as potential sites for crystallization and agglomeration to take place. This then causes the pour point of the crude oil to increase.

### Cold finger test

**Fig. 5:**
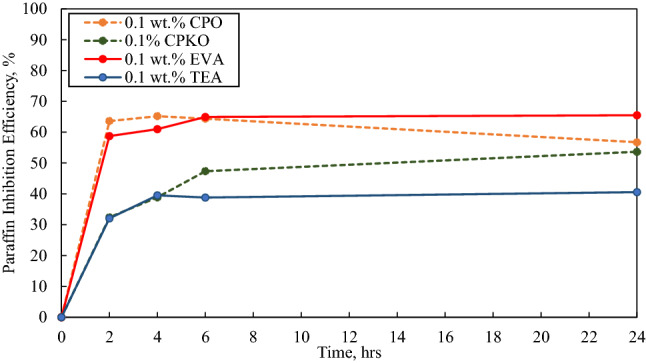
Paraffin inhibition efficiency (PIE) against deposition time in the presence of 0.1 wt.% additives under dynamic conditions

**Fig. 6:**
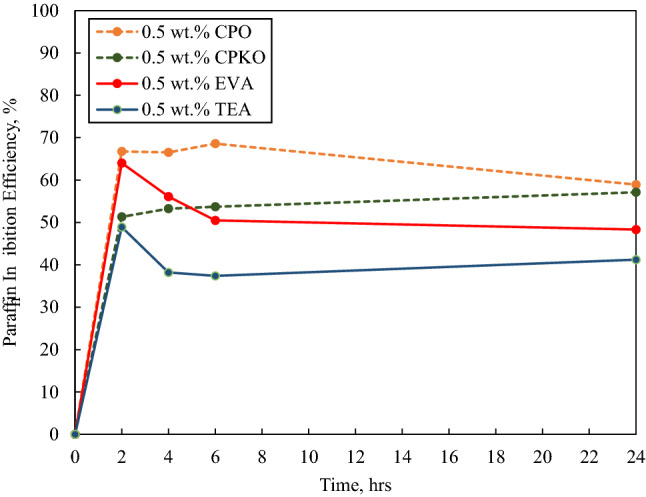
Paraffin inhibition efficiency (PIE) against deposition time in the presence of 0.5 wt.% additives under dynamic conditions

**Fig. 7:**
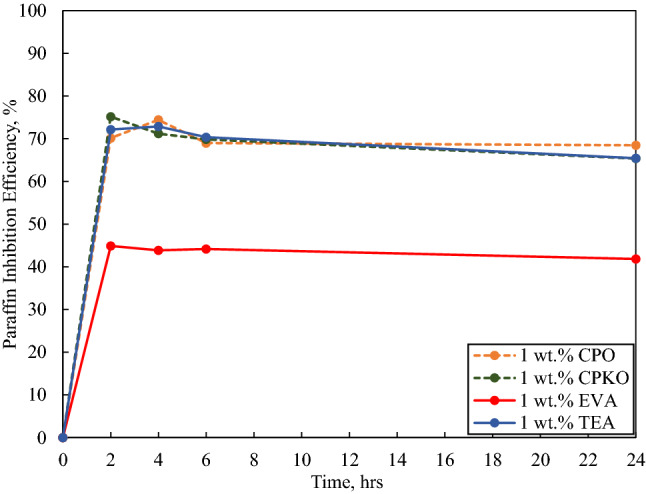
Paraffin inhibition efficiency (PIE) against deposition time in the presence of 1 wt.% additives under dynamic conditions

**Fig. 8:**
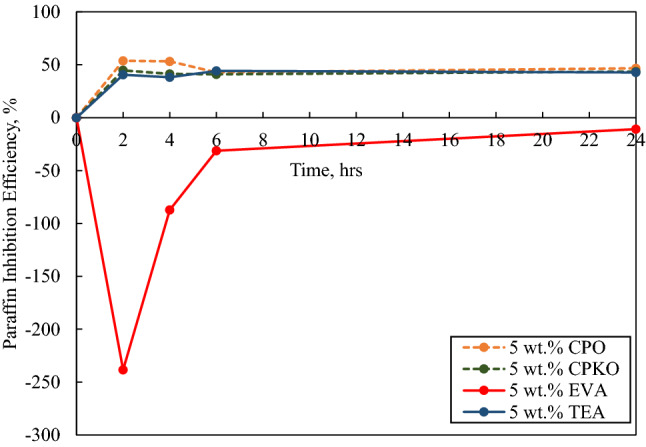
Paraffin inhibition efficiency (PIE) against deposition time in the presence of 5 wt.% additives under dynamic conditions

**Fig. 9:**
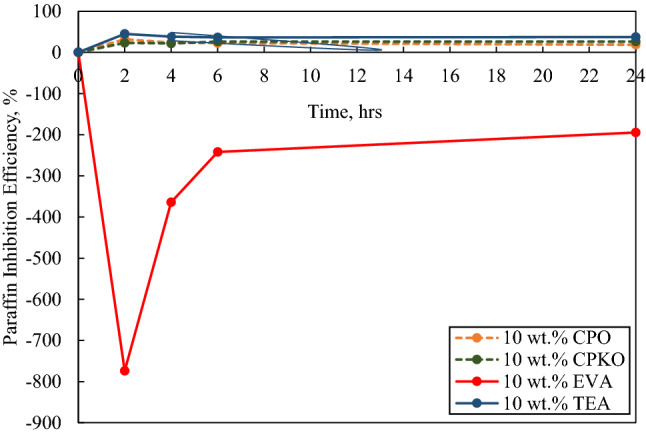
Paraffin inhibition efficiency (PIE) against deposition time in the presence of 10 wt.% additives under dynamic conditions

As discussed in the experimental section, at the end of the experiments the PIE was obtained and plotted against the deposition time as illustrated from Figs. 5, 6, 7, 8 and 9.

The mass of the wax deposited under dynamic condition was obtained using Eq. 2.

2 $$W_{{{\text{dep}}}} = W_{{\text{t}}} - W_{{\text{b}}}$$

where *W*_dep_ is the mass of wax deposited, *W*_b_ is the initial mass of the beaker, and *W*_t_ is the mass of the beaker and the scraped off deposited wax.

From the results for the dynamic deposition test, it can be noticed that the initial mass of the deposited wax increases as the deposition time increases to the 6th hour and then the rate of wax deposition decreases as the experiment approaches the 24th hour both in the presence as well as the absence of additives. This deposition trend was is caused due to the time the cold finger is in contact with the crude oil, leading to an increase in heat loss as well as lowering the bulk oil temperature leading to wax precipitation and deposition. However, the deposition trend is not linear due to the depletion effect. It is important to comprehend that as the experiment was conducted at a laboratory set up, there is a constant amount of sample used, and no fresh sample was supplied after each reading, causing wax depletion to occur as time increases (Kelechukwu et al. 2010; Misra et al. 1995). This assumption will not be applicable in a real oilfield pipeline, as there is a continuous supply of fresh crude oil flowing from the reservoir into the pipeline which is sufficient to make the depletion effect negligible (Bott and Gudmundsson 1977; Cole and Jessen 1960).

Besides, in the case of 0.1 wt.%, 0.5 wt.% and 1 wt.% inhibitor concentrations as portrayed in Figs. 5, 6 and 7, the highest wax deposited under dynamic condition was in the absence of inhibitor at 23.313 g at the 24th hour portraying that all additives possess the ability to inhibit the interlocking of crystal wax network and hence obstructing deposition at concentrations below 1 wt.% (low concentration).

#### In the presence of 0.1 wt.% additives

Figure 5 shows the paraffin inhibition efficiency (PIE) against deposition time in the presence of 0.1 wt.% concentration of inhibitors under dynamic conditions.

From Fig. 5, all the inhibitors at 0.1wt.% concentration have a positive PIE, indicating wax inhibition. The most effective of among the inhibitor tested is 0.1 wt.% EVA having an average PIE of 62.541%, followed by 0.1 wt.% CPO at 63.552%, then 0.1 wt.% CPKO at 43.065% and lastly 0.1 wt.% TEA at 37.734%. Moreover, the PIE of all the additives can be seen to deteriorate slightly after the 6th hour. This is due to the effect of wax ageing and depletion effect which has a slight effect on the additive’s effectiveness in inhibiting wax deposition at high residence time.

Furthermore, when observing the physical appearance of the deposited wax throughout the 24 h experiment, it should be noted that the all the samples tested except EVA deposited wax was slightly hard, compact, became denser and thicker as the time increases. This may be due to the effect of wax ageing as well as the presence of shear stripping where in terms of wax ageing the long chain paraffin molecules in the bulk oil continuously diffuse into the deposited wax layer on the cold finger probe. This then prompts the higher carbon number molecules to diffuse into the wax layer while the lighter carbon number molecules to diffuse out. This causes the deposited wax to have an increase in solid fraction making it denser and stiff which then increases in hardness as the time increases (Lim et al. 2018; Paso and Fogler, 2004; Quan et al. 2015). Meanwhile, the effect of shear stripping causes the weaker and softer deposited wax to detach and suspend in the crude oil (Hao et al. 2019; Leiroz & Azevedo 2005).

In the presence of 0.1 wt.% EVA however, it was found that the wax deposited was brittle and required relatively low amount of force to scrap off the cold finger probe. This characteristic of EVA is due to the fact that EVA which comprises of methyl as well as methylene groups which contain dual active oxygen atoms displays a strong van der Waals interaction between the hydrogen atoms in the crude oil. This then leads to increase solubility and the reduction of the wax gel strength causing a decline in wax deposition (Ashbaugh et al. 2005; Machado et al. 2001).

#### In the presence of 0.5 wt.% additives

Figure 6 portrays the paraffin inhibition efficiency (PIE) against deposition time for the 0.5 wt.% concentration of the additives under dynamic conditions.

According to Fig. 6, in the presence of 0.5 wt.% additives, the PIE against deposition time portrays that all the additives used have a positive PIE, implying wax inhibition. The highest PIE among the additives used was in the presence of 0.5 wt.% CPO at an average PIE of 71.170%, then 0.5 wt.% CPKO at an average PIE of 53.862%, followed by 0.5 wt.% EVA at 54.749% and lastly 0.5 wt.% TEA at 41.430%. The PIE of all the additives also follows the deterioration pattern as seen in the presence of 0.1 wt.% additive concentration. In addition, when comparing the PIE of the additives at 0.1 wt.% concentration (Fig. 5) and 0.5 wt.% concentration (Fig. 6), the PIE of CPO, CPKO and TEA can be seen to increase as the concentration increases but the PIE of EVA decreases. This implies that the increase of EVA concentration above 0.1 wt.% does not indicate higher inhibition, and the critical concentration of the additive to be the most efficient is between 0.1 wt.% and 0.5 wt.% concentration under dynamic conditions.

In addition, the physical appearance of the deposited wax throughout the 24 h experiments in the presence of 0.5 wt.% concentration of the additives was similar to the physical appearance pattern that was observed in the presence of 0.1 wt.% concentration of the additives.

#### In the presence of 1 wt.% additives

Figure 7 illustrates the paraffin inhibition efficiency (PIE) against deposition time in the presence of 1 wt.% additive under dynamic conditions.

When 1 wt.% concentration of the additives was used, the PIE against deposition time as illustrated in Fig. 7, portrays that all the additives used have a positive PIE, indicating wax inhibition occurred. The highest PIE among the additives used was in the presence of 1 wt.% CPO at an average PIE of 70.504%, then 1 wt.% CPKO at an average PIE of 70.387%, followed by 1 wt.% TEA at 70.198% and lastly 1 wt.% EVA at 43.688%. The PIE of all the additives also follows the deterioration pattern as seen in the presence of 0.1 wt.% as well as 0.5 wt.% additive concentration. Moreover, when comparing the PIE of the additives at 0.5 wt.% concentration (Fig. 6) and 1 wt.% concentration (Fig. 7), the PIE of CPO, CPKO and TEA can be seen to increase as the concentration increases but the PIE of EVA decreases drastically. This implies that the increase of EVA concentration above critical concentration of the additive can actually cause the performance of the additive to deteriorate. This result was also observed by other researchers and will be discussed in the later part of this section (Lim et al. 2018; Soni et al. 2005; Zhang et al. 2008).

Additionally, the physical appearance of the deposited wax of CPO, CPKO and TEA throughout the 24 h experiment in the presence of 1 wt.% concentration of the additives was softer and easier to be remove as compared to 0.1 wt.% and 0.5 wt.% concentration but became more compact and stiffer as the deposition time increases. However, the wax deposited in the presence of 1 wt.% EVA was unique compared to the previous concentration (0.1 wt.% and 0.5 wt.%) tested. The wax deposited was much harder and denser which made it difficult to scrap off as compared to the brittle and easy to scrap wax deposited at the lower concentration.

#### In the presence of 5 wt.% additives

The paraffin inhibition efficiency (PIE) against deposition time in the presence of 5 wt.% additive under dynamic conditions is portrayed in Fig. 8.

According to Fig. 8, the PIE against deposition time in the presence of 5 wt.% concentration portrays that 5 wt.% CPO, 5 wt.% CPKO and 5 wt.% TEA have a positive PIE at an average of 49.142%, 42.663% and 41.424% illustrating that wax inhibition occurred. While when 5 wt.% EVA was utilized, the PIE was negative at an average PIE value of − 91.161%, indicating that the presence of high concentration of 5 wt.% EVA induces further wax deposition. This is due to the possibility that at high concentrations, the EVA additives are behaving as supplementary suspended particles in the crude oil which deteriorates the efficiency of the polymer to inhibit wax deposition. In addition, when comparing the PIE in the presence of 1 wt.% inhibitor concentration, the PIE of 5 wt.% inhibitors is relatively lower, indicating that the additives function best above 1 wt.% and below 5 wt.% concentration.

In terms of the physical appearance of the deposited wax of CPO, CPKO and TEA throughout the 24 h experiment in the presence of 5 wt.% concentration of the additives was soft and easy to be remove but became more compact and stiffer as the deposition time increases. However, the wax deposited under dynamic conditions in the presence of 5 wt.% EVA was similar to the wax deposited in the presence of 1 wt.% EVA but harder and denser which were harder to scrap off.

#### In the presence of 10 wt.% additives

Figure 9 portrays that mass of wax deposited against deposition time in the presence of 10 wt.% additives.

Based on Fig. 9, the positive PIE of 10 wt.% CPO, 10 wt.% CPKO and 10 wt.% TEA portrays that the additives were still effective at inhibiting wax deposition at high concentrations. The most effective inhibitor was 10 wt.% TEA at an average PIE of 39.391%, second is 10 wt.% CPKO at average PIE of 24.439%, followed by 10 wt.% CPO at average PIE of 24.256% and finally 10 wt.% EVA having an average PIE of − 393.565%, respectively. It can be observed in the presence of 10% EVA, the PIE of the additive decreases to − 773.98% at the 2-h interval, then increases to − 194.57% at the 24-h interval. The rapid decrease in the PIE at the 2-h interval was due to EVA at high concentrations, acts as potential sites for wax crystals to agglomerate, thus decreasing the PIE. Where else, the increase in PIE afterwards, was due to the depletion effect. It is essential to understand that the experiment was conducted at a laboratory set up which has a defined amount of sample used, and there was no fresh sample that was added after each read. Therefore, the depletion of wax will occur as time increases, and there will be insufficient wax to deposit out of the crude oil and onto the cold finger probe. However, this assumption will not be applicable in a real oilfield pipeline, as there is a continuous supply of fresh crude oil flowing from the reservoir into the pipeline which is sufficient to make the depletion effect negligible.

Furthermore, the physical appearance of the deposited wax of CPO, CPKO and TEA in the presence of 10 wt.% concentration was soft and easy to be removed as observed in 0.5 wt.%, 1 wt.% and 5 wt.% concentration. However, the wax deposited in the presence of 10 wt.% EVA was extremely hard and compact as compared to 5 wt.% EVA. To better understand, the effect of the additives in various concentration tested, the graph of average paraffin inhibition efficiency against time was plotted and is illustrated in Table 4.

**Table 4:** Average paraffin inhibition efficiency (PIE) of additives tested under dynamic conditions

Additive	Average PIE
0.1 wt.% CPO	62.480
0.5 wt.% CPO	65.216
1 wt.% CPO	70.504
5 wt.% CPO	49.142
10 wt.% CPO	24.256
0.1 wt.% CPKO	43.065
0.5 wt.% CPKO	53.862
1 wt.% CPKO	70.387
5 wt.% CPKO	42.663
10 wt.% CPKO	24.439
0.1 wt.% EVA	62.541
0.5 wt.% EVA	54.749
1 wt.% EVA	43.688
5 wt.% EVA	− 91.961
10 wt.% EVA	− 393.565
0.1 wt.% TEA	37.734
0.5 wt.% TEA	41.430
1 wt.% TEA	70.198
5 wt.% TEA	41.424
10 wt.% TEA	39.391

Based on Table 4, the most effective concentration of EVA was in the presence of 0.1 wt.% concentration as it has the highest average PIE among the other concentrations of EVA tested. The low concentration of EVA causes the formation of diminutive crystals which is smaller in size nevertheless higher in numbers as compared to the paraffin plate-like crystals. Many authors have agreed that introducing EVA at low concentration or dosages agglomerates with the increasing paraffin wax crystals and hinders the crystallization process. Hence, decreasing the ability of the paraffin wax crystals to link and create network among themselves. This leads to the formation of more crystals in a diminutive form, decreasing the deposition strength (Ashbaugh et al. 2005; Jafari Ansaroudi et al. 2013). In addition, in terms of molecular discussion, as ethylene vinyl acetate composes of the copolymer of vinyl acetate and ethylene. The vinyl acetate which comprises of methylene and methyl group aids in lowering the solubility because of the high polarity property. Also, the side chains of the vinyl acetate group may also contribute to the inhibition of wax crystallization and decreases the WAT as well as pour point. The two active oxygen atoms in the vinyl acetate perform a significant part to decrease the formation of wax crystals in the crystallization process which has been thoroughly discussed under the simulation results section (Kelland 2014; Ridzuan et al. 2014).

As the concentration increased, the average PIE of EVA under dynamic conditions decreased drastically especially above 1 wt.% concentration. This is due to the possibility that the EVA additives are behaving as supplementary suspended particles in the crude oil which deteriorates the efficiency of the polymer to inhibit wax deposition. This confirms that the increase in wax deposits is influenced by the concentration of wax inhibitor utilized and as the concentration reaches the optimum value, it alters the wax crystallization behavior by implementing more structures to disturb and reconcile with edge of the developing wax crystals (Machado et al. 2001; Yang et al. 2017). On top of that, the van der Waals interaction between the crude oil and wax crystals may be altered by the presence of various concentrations of wax inhibitor used which largely influences the wax solubility in the crude oil (Machado and Lucas 1999; Ridzuan et al. 2014; Zhang et al. 2008).

**Fig. 10:**
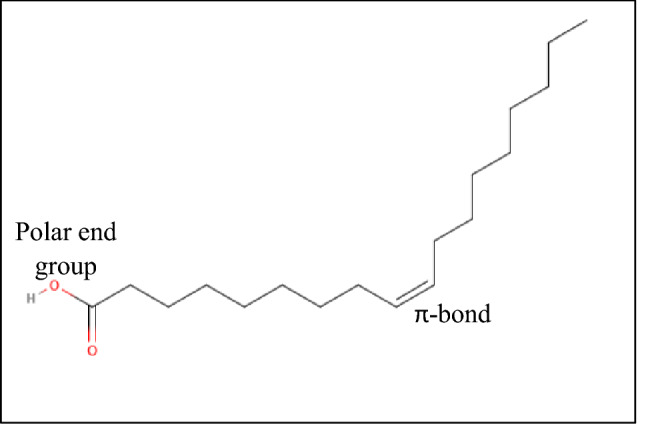
Structure of oleic acid indicating the site of interactions with the wax molecules

Besides, in the case of CPO, CPKO and TEA the most effective PIE was observed in the presence of 1 wt.% concentration based on Table 4. In terms of the palm-based additives (CPO and CPKO), the effectiveness of the additives can be attributed to the high concentration of monounsaturated molecules present (Table 5) which aids in binding the larger paraffin molecules in the crude oil, hence preventing the agglomeration as well as the deposition of wax. In addition, the effectiveness of the CPO and CPKO may also be due to the fact that the additive co-crystallizes with the paraffin molecules from the crude oil causing a decrease in wax deposition and also the pour point(Olusegun Peter Akinyemi et al. 2016). To be more precise, the unsaturated fatty acid composition of the palm oils which has a delocalized unpaired electron or the hydroxyl function over the π-orbital at the double bond (Fig. 10) may intermingle with the functionalities of the paraffin wax resulting in the reduction of the wax deposition (Akinyemi et al. 2016; Gateau et al. 2004). However, the increase of the average PIE above 1 wt.% concentration can also be attributed to the fact that the high concentration of oleic acid as well other fatty acids in the palm-based additives are acting as a potential site for wax agglomeration.

**Table 5:** Composition of fatty acids in CPO, CPKO, RSO, JSO and CSO

Fatty Acid	Lipid number	CPO, %	CPKO, %	RSO, %	JSO, %	CSO, %
Capric acid	C_10:0_	–	3.7	–	–	–
Lauric acid	C_12:0_	0.5	15.3	–	–	–
Myristic acid	C_14:0_	1.5	15.6	0.1	0	–
Palmitic acid	C_16:0_	34.5	7.8	10.2	12.33	1.45
Palmitoleic acid	C_16:1_	0.4	–	–	0.12	–
Stearic acid	C_18:0_	5.4	2	9.1	5.12	1
Oleic acid	C_18:1_	44.1	48.2	18.3	43.11	94.03
Linoleic acid	C_18:2_	12.5	2.7	38.2	39.12	2.96
α-Linolenic acid	C_18:3_	0.6	–	24.1	0	0.2
Arachidic acid	C_20:0_	0.5	–	–	0.2	–
Caproic acid	C_6:0_	–	0.3	–	–	–
Caprylic acid	C_8:0_	–	4.4	–	–	–
9,10-Dihydroxystearic	–	–	–	–	–	0.36

Meanwhile, the efficiency of TEA can be contributed to the fact that it is a cationic surfactant which have surface active agents where the surface activity depends on the nature of the quaternized amine as well as the carbon side chain length (Mahmoud et al. 2007). Yet, the average PIE above 1 wt.% concentration also increases which can also be due to the high concentration of TEA acting as potential sites for wax agglomeration.

## Conclusion

As a conclusion, the results obtained from the pour point test proves that all the inhibitors were able to reduce the pour point of the crude oil at low concentrations, however above a critical concentration, the inhibitors lose efficiency as a pour point depressant. It should be noted that the lowest pour point temperature obtained was at − 8 °C in the presence of 1% CPO and 1% CPKO, respectively. Therefore, it can be concluded that palm oil additives can be used to form a barrier to prevent the formation of wax crystal networks and hence hinder wax deposition as efficient as the commercial wax inhibitors. Meanwhile, in the case of the cold finger test, the highest PIE as well as the lowest mass of wax deposited was observed to be in the presence of 1% CPO (Average PIE = 70.504%), followed by 1% CPKO (Average PIE = 70.387%), then 1% TEA (Average PIE = 70.198%) and finally 0.1% EVA (Average PIE = 62.541%). The results not only illustrate that oleic acid or CPO and CPKO can be an excellent wax inhibitor in terms of efficiency but also portrays palm oil additives can be a potential substitute to the existing expensive and hazardous additives used in the industry. However, further investigation should be conducted on the effect of CPO and CPKO on the wax content analysis as well as the reduction of deposited wax using a flow loop apparatus before implementation in the field.

## Abbreviations

API: American Petroleum Index
CPKO: Crude palm kernel oil
CPO: Crude palm oil
CSO: Castor seed oil
EVA: Ethylene vinyl acetate
JSO: Jatropha seed oil
PIE: Paraffin inhibition efficiency
RSO: Rubber seed oil
TEA: Triethanolamine
WAT: Wax appearance temperature
